# Influence of Temporal Expectations on Response Priming by Subliminal Faces

**DOI:** 10.1371/journal.pone.0164613

**Published:** 2016-10-20

**Authors:** Swann Pichon, Raphael Guex, Patrik Vuilleumier

**Affiliations:** 1 Laboratory for Behavioral Neurology and Imaging of Cognition, Department of Neuroscience, Medical School, and Swiss Center for Affective Sciences, University of Geneva, Geneva, Switzerland; 2 Faculty of Psychology and Educational Sciences, University of Geneva, Geneva, Switzerland; 3 Swiss Center for Affective Sciences, University of Geneva, Geneva, Switzerland; University of Florida, UNITED STATES

## Abstract

Unconscious processes are often assumed immune from attention influence. Recent behavioral studies suggest however that the processing of subliminal information can be influenced by temporal attention. To examine the neural mechanisms underlying these effects, we used a stringent masking paradigm together with fMRI to investigate how temporal attention modulates the processing of unseen (masked) faces. Participants performed a gender decision task on a visible neutral target face, preceded by a masked prime face that could vary in gender (same or different than target) and emotion expression (neutral or fearful). We manipulated temporal attention by instructing participants to expect targets to appear either early or late during the stimulus sequence. Orienting temporal attention to subliminal primes influenced response priming by masked faces, even when gender was incongruent. In addition, gender-congruent primes facilitated responses regardless of attention while gender-incongruent primes reduced accuracy when attended. Emotion produced no differential effects. At the neural level, incongruent and temporally unexpected primes increased brain response in regions of the fronto-parietal attention network, reflecting greater recruitment of executive control and reorienting processes. Congruent and expected primes produced higher activations in fusiform cortex, presumably reflecting facilitation of perceptual processing. These results indicate that temporal attention can influence subliminal processing of face features, and thus facilitate information integration according to task-relevance regardless of conscious awareness. They also suggest that task-congruent information between prime and target may facilitate response priming even when temporal attention is not selectively oriented to the prime onset time.

## Introduction

Selective attention, which has traditionally been associated with modulation of perceptual processing based on dimensions such as spatial location or stimulus feature, can also operate dynamically in time [1]. Orienting attention to specific moments in time, referred here as temporal attention or temporal expectation, enhances the processing of upcoming events during this interval [1–3]. In addition to influencing motor preparation, temporal attention improves perceptual performance and reaction times in tasks requiring stimulus discrimination or identification [4, 5]. Strikingly, temporal attention has also been found to determine the extent to which subliminal stimuli influence response priming [6–8]. However, the neural underpinning of these effects remains unknown.

Behavioral studies have typically investigated the effect of temporal attention on the perception of visual stimuli flashed rapidly in a continuous sequence (e.g. rapid serial visual presentation tasks) or briefly presented below the threshold of consciousness (i.e. masked priming paradigms). For instance, generating temporal expectations regarding target onset reduces the attentional blink [9, 10]. Using a task where participants judged whether visible target numbers were greater or lower than five, Naccache and colleagues [6] showed that the effect of masked numerical primes vanished when temporal attention was focused away from the subliminal prime occurrence. Similar findings have been reported by others [7, 11]. Orienting attention in time may therefore change the dynamics and/or the strength of information processing at the supraliminal but also at the subliminal levels. Among possible mechanisms for these effects, temporal attention might produce a gain of perceptual responses [12], increase the rate of information sampling [13, 14], or anticipate the onset of perceptual evidence accumulation [15, 16]. In any case, such findings challenge the traditional view that unconscious response priming implies purely automatic and rapid feedforward processes. Rather it suggests that establishing temporal predictions through top-down attentional control may be an important factor shaping or even gating response priming [6].

Indeed, in cognitive sciences, the term automaticity is often applied to processes that are considered to be independent of top-down factors such as selective attention or task goals [17]. With regards to emotion information, in particular, processing emotionally significant stimuli (e.g., fearful expressions signaling threat) is frequently assumed to be automatic such that these stimuli are processed and memorized more efficiently [18, 19], capture attention more readily [20–23], and are perceptually more salient than neutral stimuli [24–27]. A threat advantage has been observed in various conditions where participants are not aware of the stimulus [28–34] or do not selectively attend to it [21, 35–39]. Under such conditions, threat-related responses have been measured in the amygdala, a core emotional center involved in fear and vigilance [23, 37, 40–43]. However, emotional responses may also be influenced by top-down influences related to expectations or other endogenous factors operating on sensory inputs prior to their access to consciousness [37, 39, 44]. Hence, automaticity remains an ill-defined concept that encompass several different dimensions [44, 45] and is not incompatible with modulatory effects from high-level cognitive processes—as exemplified by low-level reflexes which can be suppressed by context-dependent signals [46]. This notion is supported by the finding that temporally expected subliminal stimuli elicit significant priming effects, whereas priming is reduced for unexpected or task-irrelevant subliminal stimuli [6, 47]. Given that the status of the literature is unclear as to whether emotional subliminal stimuli receive prioritized processing independently of temporal attention, our goal was also to investigate whether, and how, temporal attention modulates the processing of masked emotional and neutral expressions.

To this aim, we used an effective masking paradigm adapted from [6]. Participants were cued (with 75% validity) to allocate their attention in time toward a visible target face embedded in a stream of successive visual noise stimuli, and performed a gender classification task as fast as possible. The target face was preceded by an invisible prime face which varied in terms of task relevance (either the same or different gender as the target) or its emotional relevance (either a neutral or fearful expression). Critically, while participants were cued to expect the target after either a short or a long time interval after the RSVP onset, the prime could appear at the predicted moment preceding the target, or at a different moment in time.

In accordance with Naccache et al. [6], we expected that temporal attention allocated to the prime-target time-window would increase response priming (i.e. decrease response times and error rate) for primes and targets which are gender-congruent, despite a lack of conscious access to the prime content. In brain data, we expect this effect to manifest by a variation of BOLD activity in regions implicated in attentional control, and possibly in regions implicated in face processing (such as the amygdala and the fusiform face area). In addition, by comparing fearful and neutral face primes, we could investigate whether or not temporal attention also influenced behavioral and brain response in the same network.

## Methods

### Participants

30 right-handed volunteers (15 females, mean age ±std 26.9±5.6, 15 males 25.8±5.6) with no history of neurological and psychiatric disease were recruited. All participants gave their informed written consent before participating in the study, which was carried out in accordance with The Code of Ethics of the World Medical Association (Declaration of Helsinki) for experiments involving humans. The study protocol, inclusion criteria, and consent procedure were reviewed and approved by the Neurosciences Cliniques Ethics Committee of the Hopital Universitaire de Genève (HUG; no 09–316). Participants were compensated for their participation in this study.

### Procedure of the fMRI task

**Fig 1:**
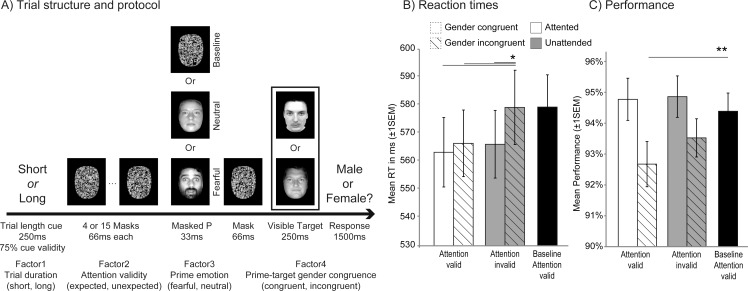
Protocol and behavioral data for the fMRI task. (A) In each trial, subjects saw a male of female target face preceded by a short or long series of noise masks (note that faces used were from the Karolinska database and were equated for luminance, they were not the example faces used in the figure). Subjects were asked to make a fast gender decision on the final target face. At the start of each trial, a word cue (the word *short* or *long*) indicated with 75% validity the time at which the target face would appear. 100 ms before the target face appeared, we presented a fearful or a neutral subliminal face (prime) or a noise mask (baseline) for 33 ms, which was both forward and backward masked by the noise masks. Gender of the subliminal face was either congruent or incongruent with the target face, while its expression was either fearful or neutral (orthogonally to other factors). (B) Response times (RTs) show that attention and gender congruence speeded correct responses. This effect did not occur on the unexpected incongruent trials (*p < .05). (C) Mean performance during target-gender decision demonstrated that more errors were made when prime gender was incongruent with target gender in expected trials (**p < .01). We have received informed consent according to Plos guidelines from the 4 individuals portrayed here.

In the scanner, subjects performed a gender recognition task on the target face appearing at the end of each RSVP (see Fig 1A). Because the efficacy of standard face-masking procedures used to probe emotion processing and the methodology used to assess prime awareness has been questioned [48, 49], we chose a novel and effective masking RSVP procedure, known to provide reliable measures of unconscious numerical priming [6]. This procedure is more stringent than common masking methods used with emotional faces, whereby emotional primes are generally presented for ~16-30ms and then backward-masked with a single neutral face [32, 50]. Here, subliminal primes were embedded in rapid succession of noise mask stimuli, presented both before and after the prime.

Subjects were asked to respond as quickly and accurately as possible using a two-buttons response box placed in their right hand. They were told that a varying number of visual noise images (either 4 or 15 different scrambled faces, 66ms each) would precede the target face (displayed for 250ms). In order to help them to respond correctly, a visual cue (duration 250ms) would appear at the beginning of each trial (the word *short* or *long*) to inform them about the onset of the target face. The cues thus instructed participants that the target would appear either shortly after trial onset (613ms i.e. 250ms+ 4*66ms masks + 33ms prime + 66ms mask) or later (1339ms i.e. 250ms+ 15*66ms masks + 33ms prime + 66ms mask). Subjects were also told that, most of the time, the cue would be informative, but that on rare occasions, it would not match the trial length. Cues were valid for 75% of the trials (baseline conditions included). Unbeknownst to the participants, a neutral or fearful prime face was presented 100ms before the target face. The prime face was made invisible by both forward masking (i.e. a series of noise masks preceding face onset) and backward masking (i.e., a final noise mask displayed for 66ms immediately after the prime and just before the target. After the target face, a response screen appeared during 1500 ms with the question “*Male or Female*?*”*. Participants had to make a response as fast and accurate as possible.

The identity of the prime face and of the target face always differed within a trial. Identities of primes within a session were never used as targets, thereby preventing the formation of any direct stimulus-response mapping. However, face identities used for primes could be used as targets in other sessions. By crossing the factors *trial length* (short vs. long), *attention validity* (targets expected vs. unexpected), *face expression* (fearful vs. neutral), and *gender congruence* (same vs. different for prime and target), we obtained 16 experimental conditions, each with 16 trials. All factors were fully counterbalanced. There were also two additional baseline conditions where a noise mask replaced the prime both for the short and long trials. Because of experimental time constraints, baseline trials included only valid cues. Each baseline condition contained 128 trials. Baseline trials were mixed with the other test conditions in each run. For each participant, we collected a total of 4 sessions comprising 128 trials each, for a total of 512 trials presented in randomized order. The total duration per session was 12 minutes.

### Objective assessment of prime awareness

Many studies have used subjective measures of prime awareness by asking participants only once at the end of the experiment whether they noticed the presence of subliminal primes, and consider negative responses as evidence for unaware perception. However, this method has been criticized [48, 49] because information from subliminal stimuli might be accessible for a short period of time only. This may be sufficient to influence behaviour, but too weak to influence conscious report. We therefore measured objective awareness by asking subjects, at each trial, whether they saw a prime and whether they could discriminate gender using a forced-choice detection task and signal detection theory methods [17, 51]. Recent studies indicated that some participants can exhibit above-chance sensitivity to emotional information at very short presentation latency (i.e. ~17ms) when prime visibility is measured ‘objectively’ [48, 49, 52], even though they report no subjective awareness of it.

Therefore, in order to evaluate prime visibility and awareness of its gender, we ran a behavioral variant of the fMRI experiment (after the fMRI experiment) asking the same participants to indicate whether a) they detected the presence of a subliminal face, b) what was their confidence level about this judgment (0–3 Likert scale), and c), when relevant, whether the subliminal face they perceived was male or female. Stimulus presentation was similar to the parameters used in the fMRI experiment.

### Stimuli

We used black-and-white pictures of female (n = 32) and male faces (n = 32) with 2 expressions (fearful and neutral) from the Karolinska Directed Emotional Faces (KDEF) dataset [53]. 20 scramble masks were created by shuffling rectangles of random size within 20 neutral faces not used in the current study. An oval mask (the shape of an average face) was applied to each image. Luminance of the resulting faces and noise masks (black oval mask excluded) was equated to a RGB value of 125 using Adobe Photoshop. We administered the behavioral protocol using E-prime (Neurobehavioral Systems, Albany, CA, USA) and programmed our script so as to insure that the RSVP of images was presented at expected time and duration. We verified the stimulation timing using a photodiode (Osram Semiconductors) placed in the beam of the scanner video projector. Stimuli were centered on the screen and each face subtended 8° of visual angle vertically and 5.5° horizontally.

### fMRI data acquisition

Gradient-echo *T*_2_*-weighted transverse echo-planar images (EPI) with BOLD contrast were acquired with a 3T Magnetom TIM Trio scanner (Siemens, Erlangen, Germany). Participants used earplugs to attenuate scanner noise. Each volume contained 36 axial slices acquired in a sequential manner (TR/TE/FA = 2100ms/30ms/80°, FOV = 205mm, resolution = 64*64, voxels size 3x3 mm with 3.2 mm thickness, distance factor 25%). We collected 1380 functional volumes for the main task in each subject, and a high-resolution *T*_1_-weighted anatomical image (TR/TI/TE/FA = 2250ms/900ms/2.6ms/9°, FOV = 256mm, resolution = 256*256, slice thickness = 1.1mm, 144 sagittal slices).

### Preprocessing of functional images

Image processing and analyses were carried out using SPM8 (Wellcome Dept. of Cognitive Neurology, London, UK). Functional images were realigned to the first volume by rigid body transformation, corrected for time differences using the middle slice in time as reference, spatially normalized to the standard Montreal Neurological Institute (MNI) EPI template, resampled to an isotropic voxel size of 2 mm, and spatially smoothed with an isotropic 8 mm full-width at half-maximum (FWHM) Gaussian kernel.

### fMRI General Linear Model analysis

We first performed standard analyses at the individual level using the general linear model (GLM) in SPM8. Each session included 18 event types as described above. Errors were modeled by a separate regressor so that only correct responses were analyzed. As accuracy was very high (94%), few responses were discarded. After model estimation, contrast images were calculated for each experimental condition, and then entered in group-level statistics.

At the group level, we used a standard repeated-measures ANOVA (flexible factorial design with 18 conditions) crossing the factors *trial length*, *attention validity*, *face expression*, and *gender congruence*, in addition to the two baseline conditions. This model was used to estimate the main effects of primes relative to baseline [all trials with face prime > all trials without face prime]. Next, contrasts that did not require a comparison with the baseline conditions were estimated using a similar ANOVA with 16 conditions (without the two baseline conditions). This avoided under-specification of the contrasts. We applied non-sphericity correction to account for variance differences across conditions. All parametric maps were rendered on the T1-weighted average brain of the group and thresholded at p < .001 uncorrected for multiple comparisons.

### Face localizer task and regions of interest (ROIs)

In addition to the priming task above, participants also underwent a standard face localizer task to identify face responsive regions, namely the amygdala and the fusiform face area (FFA) [54]. We used a 1-back task during separate blocks of neutral faces, houses, and scrambled images. There were 24 blocks, each containing 11 pictures presented for 600ms followed by a fixation cross which duration was draw from a gaussian distribution with a mean of 250ms and a standard deviation of 200ms. Subjects had to push a button whenever a stimulus was presented twice in a row. The inter-block interval was 3s and blocks were presented in random order. Maps were thresholded at p < .001 uncorrected for multiple comparisons. According to our hypothesis, the contrast faces > houses revealed activation peaks in bilateral amygdala (left: [-18–8–16], t = 5.76; right: [20–6–18], t = 5.40), and bilateral FFA (left: [-42–48–22], t = 4.25; right: [46–48–24], t = 5.65). We used these coordinates to define ROIs. Each local maximum served as the center of a 4mm sphere in order to extract beta values and perform correlation with behavioral scores using Spearman correlations. In each ROI, we averaged beta values across voxels for each contrast of interest.

## Results

### fMRI gender decision task

Globally participants performed well and made few errors (mean±SEM = 94.4%±0.4 correct). We analyzed mean reaction times (RTs) using a 4-way repeated-measure ANOVA with the factors *trial duration* (short, long), *attention validity* (expected, unexpected), prime *emotional expression* (threat, neutral), and prime-target *gender congruency* (congruent, incongruent). All error trials were discarded from the analysis. This showed a main effect of gender congruency, with significantly faster RTs when the gender of the prime and of the target were the same rather than different (564ms±12 vs. 573ms±12_,_ F_1,19_ = 5.46, p = .027). This suggests that gender congruence facilitated correct responses despite the lack of awareness of the prime (see assessment of prime awareness below). There was also a main effect of temporal attention, with faster RTs when targets appeared at expected rather than unexpected times (565ms±12 vs. 572ms±13_,_ F_1,19_ = 5.78, p = .023). Both main effects were superseded by a significant attention-by-gender congruence interaction (see Fig 1B, F_1,19_ = 4.87, p = .035). This interaction reflected the fact that, relative to the baseline condition without primes, subliminal faces facilitated performance for *unexpected* targets only when their gender was congruent, whereas performance for *expected* targets was facilitated by the preceding prime irrespective of gender congruence (see Fig 1). There was no response priming when the target was unexpected and its gender incongruent with the prime (580ms±13). In this condition, RTs were similar to baseline trials with no prime (580ms±12), and significantly longer than those in the 3 other prime conditions (all t_19_>2.8, p < .05 corrected for multiple comparisons). We found no main effect or interaction due to emotion (all p > = 0.09). There was no effect of trial duration (p = .17 for the main effect, and p > = 0.09 for all interactions with trial duration). Other two- or three-way interactions were not significant either. These results indicate that reliable priming occurred for gender-congruent trials even when attention was oriented away from prime onset, while on the contrary, no response facilitation was observed when prime gender was incongruent with target *and* attention directed away from their onset.

Although gender recognition performance was high, we conducted a similar 4-way repeated-measure ANOVA on accuracy with the factors *emotion*, *gender congruence*, *attention validity*, and *trial duration*. In line with RTs, prime-target gender congruence produced a marginal influence on performance, with more errors for incongruent than congruent conditions (93.1% vs. 94.8% correct responses, F_1,29_ = 4.06, p = .053, see Fig 1C). This trend was confirmed by posthoc tests, since error rates were higher for gender-incongruent trials than for baseline trials, when attention was oriented toward the prime appearance time (92.7% vs 94.4%, t_1,29_ = -2.8, p = 0.009), but not when attention was directed away (93.5% vs 94.4%, t_1,29_ = -1.9, p = 0.06). Accuracy in gender congruent trials did not differ from accuracy in baseline trials (both p>0.56). There was no main effect of trial duration (p = .37), emotion (p = .9), temporal attention (p = .14), or any significant attention-by-congruence interaction (p = .42). Taken together, these accuracy results suggest that, while gender congruence did not improve accuracy relative to baseline (already high overall), gender incongruence between primes and targets disrupted performance only when temporal attention was focused on the prime occurrence time.

### Objective assessment of prime awareness

**Fig 2:**
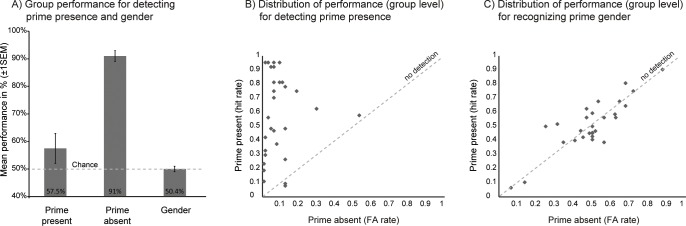
Behavioral data for the prime visibility control task. Assessment of prime visibility and gender awareness was performed after the fMRI experiment. (A) Performance of the group for detecting the presence and the content of face primes. (B) Plot of individual’s hit rate against false alarm (FA) rates for detecting the presence of the prime or (C) recognizing its gender. There was a large inter-individual variability in prime detection scores despite a general inability to consciously access gender information (note that subjects were informed of the occasional presence of primes in the behavioral test after the fMRI experiment, but not before scanning).

To evaluate prime visibility and awareness objectively, we ran a separate control experiment after the scanning session, with the same participants, display parameters and stimuli as those used during fMRI. Before starting the control experiment, subjects were made aware of the presence of prime faces in some trials. Participants were asked to indicate, for each trial, whether they saw a prime (yes-no) and assessed their level of confidence (0–3 Likert scale). In addition, when they reported having seen a prime, they were asked whether it was male or female. On average, subjects performed well to detect the absence of primes in baseline trials (Fig 2A, 91%±2 correct). However, average detection rate was at chance level to detect the presence of primes (57.5%±5.4, t_28_ = 1.37, p = .18 relative to chance). These data were further analyzed using signal detection theory methods by computing a bias-free sensitivity index (d-prime) of performance based on hit and false-alarm rates [55]. Overall, the group showed positive d-prime scores (mean:1.8±1.02, t_28_ = 9.41, p < .001 relative to 0), indicating that most subjects were able to detect the occurrence of signal above chance level (Fig 2B). Neither trial length (p = .74), gender congruence (p = .93), nor emotion (p = .74) influenced d-prime scores (please note that these scores could not be computed for the attention factor given our design).

We also estimated whether subjects’ confidence in prime detection was influenced by the same factors. Given that we considered only correct trials, we could not compute the average confidence score for each of the 16 conditions but performed paired t-tests between conditions. This analysis showed no differences in confidence depending on trial length (p = 0.1), gender congruence (p = .16), emotion (p = .43), nor attention (p = .17).

Next, we estimated individual ability to recognize the gender of the prime (Fig 2C). The group average performance was at chance (mean±SD:50.4%±5.6, t_28_ = 1.28, p = .21). This was further confirmed by d-prime scores which did not differ from zero (0.04±0.25, t_28_ = .98, p = .33). Note that d-prime scores (r = .16, p = .39) or hit rates (r = .22, p = .24) for detecting the presence of the prime were not correlated with, respectively, d-prime scores or hit-rates for recognizing the gender of the prime. Taken together, these results indicate that, despite explicit knowledge that prime faces were presented in the control experiment, participants had no conscious access to gender information.

### fMRI results: Main effects of prime and attention

**Fig 3:**
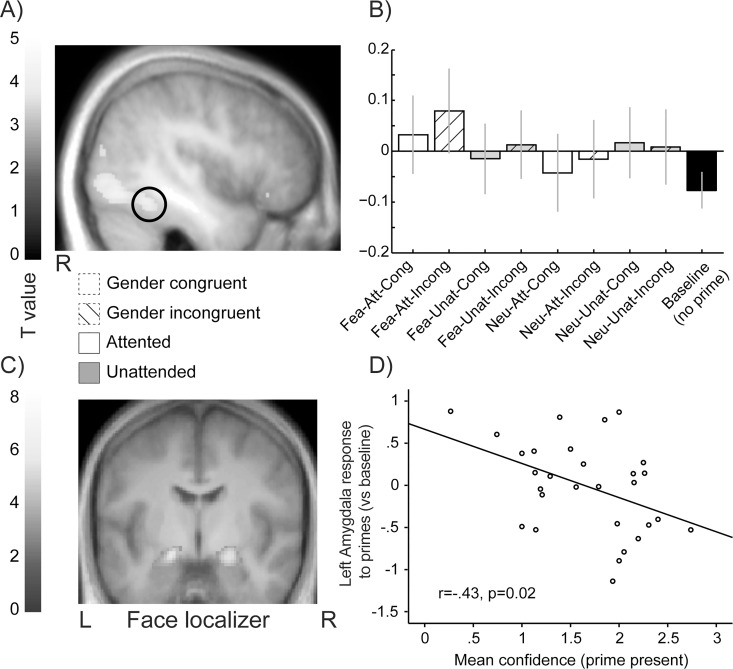
Brain response to face primes and relation to behavior. (A) Group-level contrast map (p<0.001 uncorrected) showing greater response in face-responsive cortices in trials containing a prime (versus baseline trials without prime). Note that the amygdala was not observed in this contrast. (B) Plot of parameter estimates in the right FFA as defined by the face localizer (error bars correspond to 90% confidence interval). (C) Contrast map from the face localizer showing amygdala response to faces (vs. houses). Coordinates were used to extract beta estimates in the contrast prime>no prime yet we observed no effect of emotion. (D) Interestingly, the lowest the subject’s mean confidence in prime detection, the highest the amygdala response to primes (contrast maps overlaid on the average anatomical image of the group). Abbreviations stand for trials with fearful (Fea) or neutral primes (Neu), temporally expected (Att) or unexpected targets (Unat), prime-target gender congruence (Cong) or incongruence (Incong).

Comparing trials with a prime to baseline trials with no prime yielded robust activations in face sensitive regions (Fig 3A), comprising the bilateral occipital face area (OFA) and right fusiform face area (FFA), in addition to bilateral inferior frontal gyrus (IFG BA45 & BA47). No activation was observed in amygdala, even at lower thresholds (see Table 1 for full list of activations). This result converges with the behavioral findings above to demonstrate that face primes did undergo visual processing, despite the lack of reliable conscious perception and despite the fact that subjects ignored that primes were presented during scanning.

**Table 1:** Effect of prime > baseline trials.

R/L	Anatomical region	MNI coordinates			Z value	Size in voxels
		x	y	z		
R	FFA	42	-50	-20	4.3	664
R	OFA	44	-72	-12	4.2	↓664
L	OFA	-42	-76	-16	3.7	81
R	Occipital gyrus	46	-78	-6	4.0	↓664
R	Occipital gyrus	50	-80	12	3.6	40
R	Orbitofrontal cortex (BA47)	44	26	-14	3.4	12
L	Inferior frontal gyrus (BA45)	-50	18	8	3.2	2

Height t-threshold at 3.1 corresponding to p<0.001 uncorrected. FFA: fusiform face area, OFA: occipital face area. ↓ indicates a subpeak in a bigger cluster.

**Fig 4:**
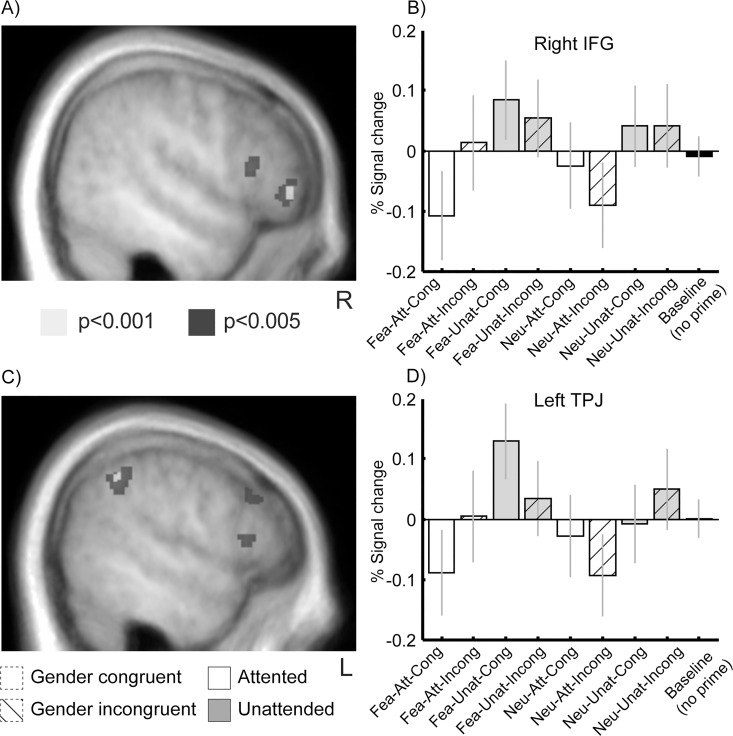
Effect of temporal attention. (A-C) Group-level contrast map and (B-D) mean group parameter estimates showing the effect of attention reorienting (i.e. unexpected > expected trials) in right IFG and left TPJ (same conventions as in Fig 3).

Next, we identified brain regions differentially engaged in trials where targets were unexpected, relative to expected (invalid temporal cueing; see Fig 4), across all prime conditions. This contrast revealed significant activations in the right IFG (xyz = [52 42–4], Z = 3.3 and [40 34 6], Z = 3.3) and a cluster in left angular gyrus / temporo-parietal junction (TPJ, [-56–50 42], Z = 3.4), two regions known to be critically implicated in response inhibition such as no-go tasks [56] and attention shifts induced by exogenous cues [57]. These effects accord with a need to reorient attention in time when face targets appeared after a temporal interval unpredicted by the initial temporal cues (i.e. earlier or later than expected) [3], and in turn confirm that our participants used these temporal cues to prepare their response to upcoming targets. The opposite comparison (expected > unexpected) yielded activations in right ventral putamen ([18 16–12], Z = 3.4), left posterior insula ([-38–28 2], Z = 4.3), left parietal operculum (i.e. OP4 at [-52–2 8], Z = 3.4), and left lateral occipital cortex ([-42–84 18], Z = 3.3).

**Fig 5:**
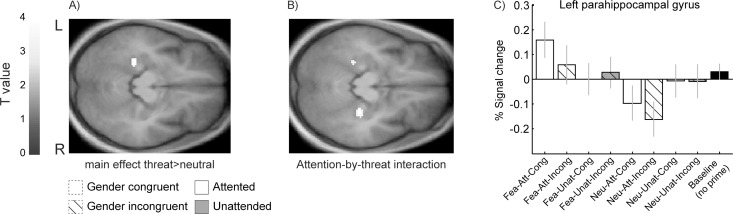
Effect of temporal attention on fearful primes. (A) Group-level contrast map (p<0.001 uncorrected) showing greater responses in left parahippocampal gyrus on trials with fearful prime (vs neutral). (B) Contrast map and (C) group parameter estimates illustrating the attention-by-emotion interaction in bilateral parahippocampal gyrus, indicating that temporal attention specifically enhanced the processing of fearful primes relative to neutral or unattended primes (same conventions as in Fig 3).

To determine the main effect of emotion, we then contrasted trials with fearful primes against neutral ones (see Table 2 and Fig 5). This yielded selective activations in the left parahippocampal gyrus (PGH). Contrary to our predictions, no differential activation was found in face and emotion responsive regions (i.e., amygdala and FFA). The attention-by-emotion interaction (masked inclusively by the contrast [threat>neutral]) also revealed activations in bilateral PGH, indicating that activity in this region was further enhanced when fearful primes were temporally attended (Fig 5). Again, no interaction was found in face-responsive regions, including amygdala. No correlations were observed between behavioral scores (d-prime measures and confidence ratings) and beta parameters extracted from PGH from the contrast threat > neutral primes (all p’s > 0.24, d-prime prime presence: left/right PGH: r = .08/0.12, confidence prime presence: left/right PGH: r = -.18/-0.18, d-prime gender: left/right PGH: r = .22/0.17).

**Table 2:** Effect of fearful primes and of temporal attention.

		fearful > neutral primes					Interaction attention*emotion masked inclusively with threat>neutral				
** **	** **										
R/L	Anatomical region	MNI coordinates			Z value	Size	MNI coordinates			Z value	Size in voxels
		x	y	z			x	y	z		
R	Parahippocampal gyrus						28	-34	-16	3.63	7
L	Parahippocampal gyrus	-30	-36	-18	3.5	41	-30	-40	-18	3.77	130

Height t-threshold at 3.1 corresponding to p<0.001 uncorrected

We finally probed for the neural signature of the gender-congruence effect observed between prime and target in the behavioral data. Contrasting gender-incongruent to gender-congruent trials yielded activations in several regions associated with attention, motor control, and response monitoring, including anterior insula, anterior cingulate cortex (ACC), dorsal prefrontal cortex (superior frontal sulcus), and intra-parietal sulcus (IPS, see Table 3, S1 Fig and Fig 3). Neither the reverse contrast of congruent versus incongruent trials, nor the attention-by-gender congruence interaction yielded significant activation. These results suggest that gender incongruence, between the invisible prime and the visible target, induced a distinctive recruitment of motor and attentional control due to response conflict, despite weak differences in perceptual processing observed in visual cortex.

**Table 3:** Effect of attention and prime-target gender congruence.

		Main effect gender incongruent > conrugent primes				
	
R/L	Anatomical region	MNI coordinates			Z value	Size
		x	y	z		
**Gender incongruent > congruent**						
L	Anterior insula	-28	30	2	4.1	139
L	Superior frontal sulcus	-28	28	28	3.9	39
R	Anterior cingulate cortex / pre-SMA	10	6	52	3.6	58
L	Intra-parietal sulcus	-32	-52	46	3.2	6
R	Cerebellum	34	-56	-34	3.2	11
L	Cuneus	-12	-80	22	3.4	13
R	Cuneus	18	-40	-34	3.2	5
R	Precuneus	6	-68	58	3.6	20
**Gender congruent > incongruent**		None				
**Unexpected Incongruent versus others conditions**						
L	Anterior insula	-34	30	-6	3.3	4
L	Dorsolateral prefrontal cortex	-50	24	38	3.5	37
R	Dorsolateral prefrontal cortex	58	20	34	3.2	1
R	Intra-parietal sulcus	40	-50	50	3.3	17
L	Intra-parietal sulcus	-34	-54	44	3.1	1
L	Fronto-polar cortex	-30	62	14	3.7	43
**Others conditions versus unexpected incongruent**						
R	Fusiform gyrus	34	-66	-4	3.6	15
R	Angular gyrus / temporo-parietal junction	40	-36	22	3.3	3
L	Heschl gyrus	-44	-22	4	3.3	10

Height t-threshold at 3.1 corresponding to p<0.001 uncorrected

**Fig 6:**
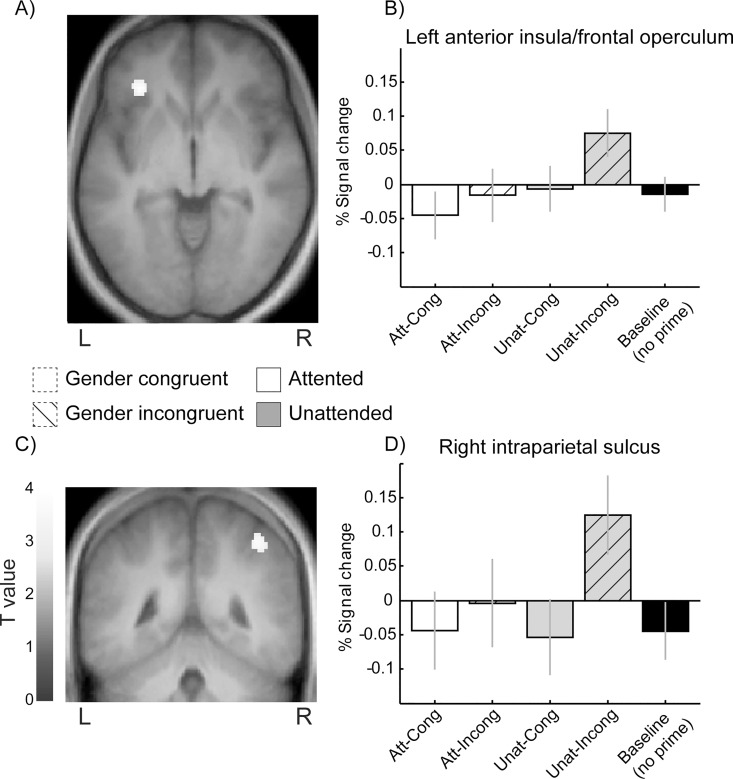
**Effect of temporal attention on prime-target gender congruence** (A) Group-level contrast showing the combined effects of gender incongruence and unexpected targets in (A) left anterior insula and (C) right IPS. (B-D) show corresponding mean group parameter estimates (same conventions as in Fig 3). Note that this effect was not present when incongruent primes were attended and that the pattern of activity mirrors the RT data.

Furthermore, to pinpoint brain regions involved in the slowing of RTs on incongruent trials which was selectively observed for unexpected incongruent targets (see Fig 1B), we directly contrasted the latter trials against all three other prime conditions (i.e. crossing the factors attention and gender congruence, see Fig 6 and Table 3). This contrast confirmed stronger activations in left anterior insula, bilateral dorsolateral prefrontal cortex (dlPFC) and IPS, as well as fronto-polar cortex. We found no significant attention-by-gender congruence interaction. These results suggest that the effects of gender incongruence and unexpected temporal onset were independent and additive.

We also sought to identify brain regions showing the same response priming benefits as reflected in RTs, by computing the reverse contrast between all conditions with facilitated responses, relative to trials with unexpected incongruent targets (see Fig 1B). This contrast revealed significant activation in the right fusiform gyrus and the right TPJ (see Table 3). This suggests reduced visual processing and less efficient attentional orienting when gender-incongruent primes precede unexpected targets.

### fMRI results: Correlation of behavioral performance with face-selective activations

Since visual awareness might modulate amygdala response to subliminal emotional faces [49], we examined the relation between neural activity (beta parameters) measured from emotion- and face-responsive ROIs (as defined by the face localizer, see methods) and three indices of behavioral performance in the post-scanning awareness test: 1) prime detection abilities (using hit rate for correctly reporting prime presence); 2) prime gender discrimination (correct identification of face gender) and 3) confidence scores (for correctly detected primes). These analyses revealed that increased left amygdala response to trials with a prime (versus no prime) was *negatively* correlated with confidence (r = -.43, p = .02; see Fig 3B), but also marginally negatively correlated with the individual ability to detect prime presence (r = -.35, p = .06). Thus, higher amygdala activity appeared related to lower confidence and lower prime detection. No effect was observed separately for fearful or neutral faces (all p>.2). We found no correlations with FFA.

## Discussion

In the present study, we tested the hypothesis that subliminal perception is influenced by temporal attention [6], and investigated the neural underpinnings of such effects in a face categorization task. By using fMRI together with an effective masking paradigm, we manipulated temporal expectations and the content of primes along both gender and emotional expression of faces.

Our results converge with earlier behavioral findings and show that response priming is influenced both by congruent task-relevant features and by temporal attention, despite subjects’ inability to identify the prime’s gender. At the neural level, we find reduced activation in the face-responsive fusiform cortex for task-relevant congruent features and/or when attention was temporally focused on primes. Conversely, task-incongruent features that were not attended produced distinctive activations in brain regions involved in response conflict and attention reorienting. Our results thus offer a nuanced perspective according to which temporal attention can influence, but does not dictate, subliminal perception of visual (face) information. Crucially, gender-incongruent trials appearing at unexpected time produced distinctive activations in fronto-parietal regions involved in attention reorienting and reduced activation of face-responsive areas in fusiform cortex. Altogether this pattern uncovers neural substrates in both the attentional and perceptual systems that mediate the effects of temporal expectations and subliminal primes on visual processing.

By contrast, although subliminal prime faces did activate extrastriate areas relative to the no-prime baseline condition, their emotional content did not produce strong visual or attentional effects, including in face-responsive ROIs (fusiform cortex and amygdala). However, we found that emotion interacted with temporal attention to activate the parahippocampal gyrus (PGH) bilaterally, which was sensitive to the expression of face primes only when attended.

Our results accord with the view that response priming due to subliminal stimuli can be facilitated by temporal attention [6] or sensitization by prior task-set which enhances cognitive processes congruent with the task [58, 59]. Enns et al. [47], for instance, have shown that congruency effects between target faces and primes that varied in emotion, gender, and race, depended on which feature was relevant to the categorization task the participants were currently engaged in. Response priming was present when the feature shared between the prime and the target was task relevant, but was abolished when the feature became task irrelevant or unexpected [47, 60]. Response priming is also reduced by attention depletion [61]. Likewise, non-conscious numerical priming is enhanced when primes are temporally attended (i.e. appearing close to expected target onset) but suppressed when prime onset time is unexpected [6]. Together, these findings suggest that “automatic” priming effects are at least partly shaped by the operation of dynamic top-down attentional processes.

Our results also extend previous results by revealing different effects of temporal attention as a function of prime congruency. In our study, priming by gender-congruent faces occurred regardless of temporal attention, whereas priming by gender-incongruent faces occurred only in valid/expected trials (i.e. when attention was temporally directed to the upcoming target). A first possible interpretation is that gender-congruent features could benefit from the sensitization of task-related processing pathways even when they do not overlap with specific target features [11, 47, 58, 59], and thus still produce response priming despite suboptimal temporal attention. This account would accord with the maintenance of response priming for unexpected but congruent trials, unlike for unexpected incongruent trials. Another interpretation is that this RT pattern might result from the mere presence of a prime (versus baseline) that could act as a non-specific alerting signal. However this interpretation does not account for the gender congruency effect observed during unexpected trials. If primes were simply acting as alerting signals and their content was not extracted subliminally, then we should observe similar slowing of RTs in all unexpected trials, no matter whether gender between the prime and the target is congruent or not. Second, an alerting effect alone does not account either for the deleterious effect on error rates during *expected* incongruent trials. Instead, this effect would accord with the interpretation that temporal attention heightens the processing of the subliminal cue.

Task-relevant unconscious signals may not only activate perceptual pathways, but also influence brain regions responsible for implementing specific cognitive processes. For example, participants instructed to perform a semantic or a phonological task based on a visual cue, show decreased performance when an unconscious cue signaling the alternative task is presented at the beginning of a trial [62]. These effects are paralleled by up- or down-regulation of brain networks associated with each task. Similarly, unconscious no-go signals may slow response time and increase activity of inferior frontal cortex and pre-SMA, a brain network involved in response inhibition [63] that was also activated during unexpected incongruent trials. Our fMRI results converge with these findings by showing that several prefrontal regions associated with response conflict and attention control were more engaged when gender between primes and targets was incongruent (see S1 Fig). These effects involved the right dorsolateral prefrontal cortex, ACC and pre-SMA, as well as insula and IPS. The same regions were even more engaged by unexpected incongruent trials relative to other conditions (Fig 6).

The latter network is likely to reflect the conflict induced by the concomitant activation of two divergent motor responses by the prime and the target. Pre-SMA is involved in response plan updating when a response switch is required [64, 65]. ACC and insula are also implicated in error monitoring and awareness [66, 67]. IPS and dlPFC are key regions of the dorsal goal-directed attentional system [57]. IPS itself has been associated with selective attention orienting [68] in both time and space [1, 69]. Altogether, these regions are often reported in tasks involving response conflict and making greater demands on executive control [70]. Here, remarkably, the recruitment of these networks was triggered by masked primes without conscious detection of gender incongruence, indicating that modulation of cognitive control can arise unintentionally, without conscious perception of the stimulus generating response conflict. Furthermore, the fact that this executive control network showed lower activity during unexpected but gender-congruent trials further suggests that subliminal face information could facilitate gender decisions and dampen the cost of suboptimal temporal attention.

We found no significant behavioral effect of emotion on response priming. It is possible that behavioral priming might occur mainly when emotion is task-relevant [71]. Therefore, the lack of emotional effect on behavioral performance in our paradigm might reflect the fact that gender and emotion processing rely on independent processes [72], and/or that emotion expression was never task-relevant during the whole experiment, precluding any sensitization of emotion pathways to facial expressions [47]. In line with behavioral results, no differential brain responses were observed in visual or limbic areas for subliminal fearful faces, in contrast with other studies where emotional faces were presented in visible and invisible conditions [49, 73]. Further research is needed to determine whether emotion might have produced a stronger impact on behavior and/or brain activity if subjects had performed a decision for which emotion was task-relevant, either on the same trials or during the same experiment.

The only brain region reactive to subliminal fearful primes was the parahippocampal gyrus (PHG). This area showed further increased activity for invisible fearful faces when temporal attention valid, but it was not influenced by gender congruence. Medial temporal structures, including PGH, are known to subserve episodic memory [74–77]. Emotional items are usually better encoded and recognized than neutral ones [19], an effect mediated by modulatory influences from the basolateral amygdala on medial temporal structures, including the PGH [78, 79]. Despite the weak effect of emotion in our task, subliminal fearful primes might nonetheless receive residual processing and modulate affective memory processes in PHG. Since face identities used as subliminal primes were seen as neutral targets in different blocks, it is possible that some memory traces or associations created in PHG by the visible faces were then reactivated by the same faces when they were presented subliminally and associated with emotional meaning. Moreover, access to these memory associations might be enhanced with attention, accounting for the interaction effect seen in PHG. Alternatively, recent fMRI evidence suggests that PGH participates in object ensemble representation (e.g. leaves on a tree crown). Unlike other regions involved in object recognition, PGH/PPA shows fMRI adaptation to different objects of the same category [80] and might compute summary statistics for this ensemble [81, 82]. This proposition offers an alternative interpretation of our results, namely that PGH may accumulate information from faces appearing sequentially in time, with stronger adaptation (repetition suppression) when these faces share congruent and/or neutral features than when they do not, for instance when a fearful prime precedes a neutral target. Moreover, PGH activity was maximal for emotional primes and minimal for neutral primes when these appeared during attended time intervals, consistent with the observation that fMRI adaptation is amplified by attention [83, 84]. Interestingly, no such suppression effect was observed in PGH for gender-congruent (versus incongruent) trials, further confirming that subliminal emotion features were selectively processed in anterior temporal areas and modulated by temporal attention.

Despite the fact that activity in the amygdala was not sensitive to the different prime conditions, amygdala response to primes varied negatively according to individual confidence in prime detection ability, independently of emotion. Interestingly, Pessoa and colleagues [49] reported that amygdala activity to fearful expressions was positively related to the ability to perceive the content of the prime. Here, we ensured that the content of the prime (i.e., face gender) was not identifiable using a stringent masking procedure. Similar to Pessoa and colleagues [49], we observed no amygdala response to primes at perceptual thresholds were subjects could not access the content of the prime. However unlike their results, we observed a negative relationship between subjects’ ability to detect the presence of the prime or related confidence ratings, and the magnitude of amygdala response to primes (relative to the baseline condition). A potential explanation for the discrepency between both studies, is that our subjects remained unaware of the existence of subliminal primes during the entire fMRI experiment, whereas participants in Pessoa and colleagues [49] were explicitly prompted to detect (or guess) whether trials contained a neutral or a fearful prime. Awareness of the content of masked faces, which has been associated with increased amygdala activity, differs from the mere gist that “something has been flashed”. The latter information may not be sufficient to elicit amygdala responses or generate predictions regarding the content of the prime. Instead, higher amygdala response associated with lower confidence ratings, as observed here, accords well with the view that the amygdala is preferentially engaged by ambiguous or uncertain situations of biological relevance [42, 85].

As a limitation, we would like to mention that the use of a task which made gender—but not emotion—relevant to the decision at hand, limits our capacity to interpret whether or not emotion might have produced a stronger impact on brain and behavior if subjects had been requested to process explicitly the emotional dimension of the target stimulus. More research is needed to fully clarify this question.

## Conclusion

Altogether, our results provide new support in favor of top-down modulation of subliminal processing by attentional factors. Our findings add to recent models proposing that unconscious processes are not fully independent of selective attention [6, 11]. At the same time, they also suggest that task-congruent information between prime and target can facilitate response priming even when temporal attention is not selectively oriented to the prime onset time. These results offer a nuanced perspective according to which temporal attention can influence, but does not dictate, subliminal perception of visual (face) information.

## Supporting Information

S1 Fig Effect of prime-target gender incongruence. Group-level contrast map showing greater activity in pre-SMA and left frontal operculum/anterior insula induced by gender incongruence between prime and target (same conventions as in Fig 3). Other prefrontal areas showing similar gender incongruence effects included dlPFC, ACC and IPS (not shown here).
(TIF)
