# Supplementary figures and images for: Influence of Temporal Expectations on Response Priming by Subliminal Faces
Source: PLoS One. 2016 Oct 20;11(10):e0164613. doi: 10.1371/journal.pone.0164613 (PMC5072568; doi:10.1371/journal.pone.0164613)

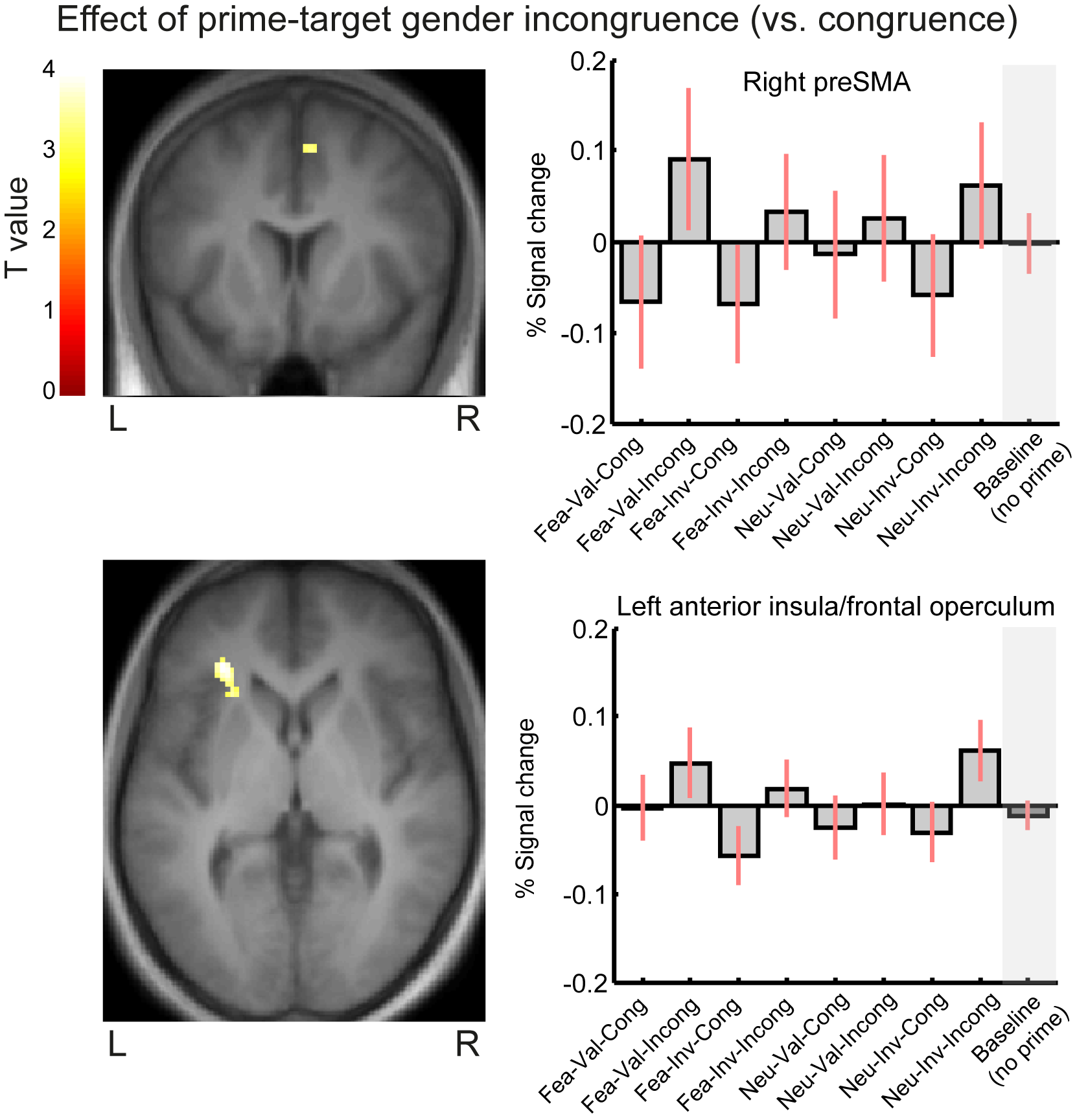

Supplement: S1 Fig — Group-level contrast map showing greater activity in pre-SMA and left frontal operculum/anterior insula induced by gender incongruence between prime and target (same conventions as in Fig 3). Other prefrontal areas showing similar gender incongruence effects included dlPFC, ACC and IPS (not shown here). (TIF) [file pone.0164613.s001.tif]
